# Severe COVID-19 Infection during Pregnancy Requiring ECMO: Case Report and Review of the Literature

**DOI:** 10.3390/jpm13020263

**Published:** 2023-01-30

**Authors:** Diana Diago-Muñoz, Alicia Martínez-Varea, Esther Pérez-Sancho, Vicente Diago-Almela

**Affiliations:** 1 Department of Obstetrics and Gynaecology, Santa Lucia General University Hospital, 30202 Cartagena, Spain; 2 Department of Obstetrics and Gynaecology, La Fe University and Polytechnic Hospital, Avenida Fernando Abril Martorell 106, 46026 Valencia, Spain; 3 Department of Anesthesiology, La Fe University and Polytechnic Hospital, Avenida Fernando Abril Martorell 106, 46026 Valencia, Spain

**Keywords:** SARS-CoV-2 infection, COVID-19 vaccination, ECMO, pregnancy

## Abstract

Background: The risk of developing severe COVID-19 that requires admission to an intensive care unit (ICU) and invasive ventilation is increased in pregnant women. Extracorporeal membrane oxygenation (ECMO) has been successfully used to manage critical pregnant and peripartum patients. Case Report: A 40-year-old patient, unvaccinated for COVID-19, presented to a tertiary hospital in January 2021 at 23 weeks of gestation due to respiratory distress, cough, and fever. The patient had a confirmed diagnosis of SARS-CoV-2 with a PCR test in a private center 48 h before. She required admission into the ICU due to respiratory failure. High-flow nasal oxygen therapy, intermittent noninvasive mechanical ventilation (BiPAP), mechanical ventilation, prone positioning, and nitric oxide therapy were administered. Additionally, hypoxemic respiratory failure was diagnosed. Thus, circulatory assistance using ECMO with venovenous access was performed. After 33 days of ICU admission, the patient was transferred to the internal medicine department. She was discharged 45 days after hospital admission. At 37 weeks of gestation, the patient presented active labor and underwent an uneventful vaginal delivery. Conclusions: Severe COVID-19 in pregnancy may lead to the requirement for ECMO administration. This therapy should be administered in specialized hospitals using a multidisciplinary approach. COVID-19 vaccination should be strongly recommended to pregnant women to decrease the risk of severe COVID-19.

## 1. Introduction

In the pandemic associated with infection with SARS-CoV-2 and its related clinical disease COVID-19, over 650 million people have been infected, and over 6,6 million have died worldwide as of 14 December 2022 [1]. Almost 4–6% of women in the childbearing age group, who are critically ill with COVID-19 and admitted to the intensive care unit (ICU), are pregnant [2].

The vast majority of cases of SARS-CoV-2 infection in pregnant women are asymptomatic or present mild influenza-like symptoms, similar to the general population. Nonetheless, the risk of developing severe COVID-19 that requires admission to the ICU and invasive ventilation is increased in pregnant women [3]. Moreover, pregnant women with comorbidities such as obesity, diabetes mellitus, and chronic hypertension are at a higher risk of severe COVID-19 [4]. In severe cases with no improvement after drug therapy and mechanical ventilation, extracorporeal membrane oxygenation (ECMO) is required [4]. This paper describes a case report of a woman with severe COVID-19 during the second trimester of pregnancy who underwent successful treatment with venovenous (VV) ECMO, and provides a review of the literature regarding the effectiveness of ECMO in pregnant patients with severe COVID-19.

## 2. Case Report

A 40-year-old patient, gravida three, para two, presented to the emergency department of La Fe University and Polytechnic Hospital (Valencia, Spain) in January 2021 at 23 weeks of gestation due to respiratory distress, cough, and fever. The patient had a confirmed diagnosis of SARS-CoV-2 with a polymerase chain reaction (PCR) test performed in a private center 48 h before. The patient had a known bicornuate uterus. She underwent one vaginal delivery at term and one subsequent preterm delivery at 28 weeks of gestation. The preterm newborn presented perinatal mortality of unknown origin in another hospital. The current pregnancy had a low-risk first-trimester screening for chromosomal abnormalities and a normal morphological ultrasound at 20 weeks of pregnancy.

At the emergency department, the patient manifested pleuritic chest pain, anosmia, dysgeusia, tachypnea, and dyspnea with minimal effort. Arterial blood gas displayed respiratory alkalosis, hypoxemia, and an inspired oxygen fraction index (PAFI) of 143. Blood tests showed a reactive C-protein (CRP) of 162.2 and a D-dimer of 55.800. No other parameters were abnormal. A chest X-ray revealed extensive bilateral areas of consolidation. Thus, she was admitted to the hospital. Low-molecular-weight heparin (LMWH) of 40 mg/12 h and dexamethasone were initially administered.

**Figure 1:**
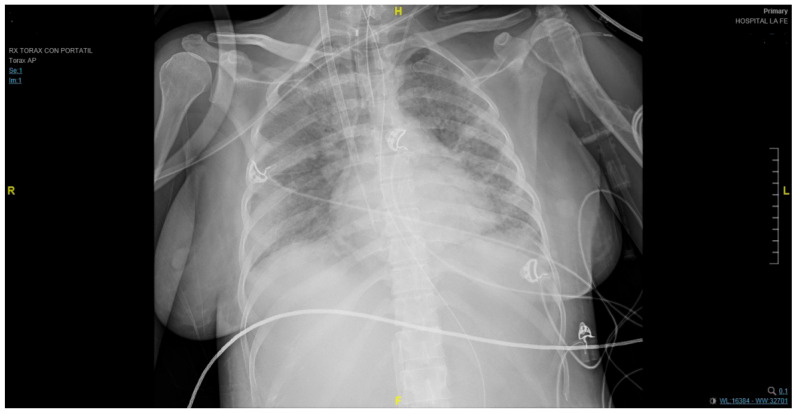
An anteroposterior chest X-ray revealed extensive bilateral areas of consolidation and the endotracheal tube at 2 cm of carina.

The patient required admission into the ICU one day after being admitted into the hospital due to respiratory failure requiring high-flow nasal oxygen therapy at the beginning. Later, intermittent noninvasive mechanical ventilation (BiPAP) was administered because of her poor clinical evolution. Nonetheless, on the fourth day in the ICU, the patient required orotracheal intubation for mechanical ventilation with a neuromuscular blockade. Functional deterioration persisted despite prone positioning (adapted to the pregnant woman’s condition) and nitric oxide therapy. Hypoxemic respiratory failure (p02 58 mmHg) was diagnosed. Thus, circulatory assistance using ECMO with venovenous access (right femoral–right jugular) under prophylactic antibiotic therapy was performed (Figure 1). During the entire hospital admission, a close pregnancy follow-up was carried out through maternal and fetal assessments that included a fetal ultrasound every 24–72 h.

ECMO began with flows of 4.2 L/min and FiO_2_ 100% (at 4 L/min in the gas blender), increasing during the first days to 5.5 L/min to achieve proper oxygenation. Lung-protective ventilation was used, with a tidal volume of 3 mL/kg weight, FiO_2_ 80%, and PEEP 10 cm H_2_O.

During the first 24 h after the implantation of the VV ECMO, anticoagulation was not used to avoid bleeding problems. After the first day, unfractionated heparin infusion was started, its administration controlled through an activated coagulation time (ACT) (160–180). In this case, as the peripheral insertion was percutaneous, the heparin administered for cannulation was not reversed.

After 15 days of ICU admission, a tracheostomy was performed, suspending the infusion of unfractionated heparin for a few hours, which could later be resumed without complications.

Seventeen days after ICU admission, sedation and the neuromuscular blockade were withdrawn. The respiratory transition was started using pressure support ventilation (PSV), concomitantly reducing the VV ECMO assistance flows.

Six days after the tracheostomy, on day twenty-one, a clear improvement was observed, and ECMO was withdrawn with PaO_2_/FiO_2_ 382 mmHg. Tolerance to oral feeding, together with enteral nutrition, was started.

At 24 days of ICU admission, the consolidation of spontaneous ventilation through tracheostomy with the active expectoration of secretions was seen. Active respiratory and motor physiotherapy were carried out.

At 32 days of ICU admission, a tracheostomy decannulation was performed without incident. Spontaneous ventilation with nasal O_2_ at 2 L/min (FiO_2_ 0.28) was started. After 33 days of ICU admission, the patient was discharged from resuscitation and transferred to the internal medicine department (Table 1 shows gasometric data during ICU admission).

Fetal lung maturation with betamethasone was carried out at 26 weeks of pregnancy. The obstetric follow-up was satisfactory. The fetal ultrasound revealed that the fetal growth and Doppler study, as well as the amniotic fluid, were within normal limits. An improvement in motor function with physiotherapy was seen. However, the patient developed bilateral external sciatic–popliteal nerve neuropathy due to prolonged rest. Forty-five days after her admission, the patient was discharged from the hospital.

At 37 weeks of gestation, the patient presented to the emergency department due to active labor. She underwent a vaginal delivery. The weight of the male newborn was 2750 g, the Apgar score was 10, arterial pH from cord blood was 7.29, and venous pH was 7.38. The patient was discharged 48 h later with a 40 mg LMWH treatment for six weeks.

At the outpatient evaluation four weeks after birth, the patient reported an improvement in muscle fatigue and weakness. Nonetheless, she described persistent dyspnea with heavy exercise. Afterward, the patient underwent a subsequent follow-up with her general practitioner.

## 3. Discussion

ECMO is an invasive support strategy for cardiac, respiratory, or combined cardiorespiratory failure when conventional treatment options have failed [5]. ECMO should be considered as salvage therapy in cases of refractory or conventional mechanical ventilation, ventilation in the prone position, and recruitment maneuvers [6].

ECMO has been successfully used to manage pregnant patients in critical condition [5,7,8]. Potential indications for ECMO in pregnant women are pulmonary embolism, amniotic fluid embolism, cardiomyopathy, and primary pulmonary hypertension with right heart failure [9]. The survival rate of pregnant women on ECMO is 75–80%, and the fetal survival is 65–70% [10]. A recent paper ensured that pregnancy can be prolonged on ECMO, delivery on ECMO can be performed safely, and ECMO can serve as a bridge to maternal respiratory recovery [11]. Moreover, Hansra et al. described a VV ECMO administration during the second trimester of pregnancy, leading to a successful pregnancy outcome [12].

Regarding severe COVID-19 in pregnant women, if it occurs during the third trimester of pregnancy, an elective preterm birth may improve the maternal condition [7,13,14]. Indeed, pregnant women with SARS-CoV-2 infection have been associated with an approximately three times higher risk of preterm birth than those without the infection due to a severe maternal condition [7,15]. Additionally, pregnant women with COVID-19 should be closely monitored, as the disease has been associated with low birthweight [13,16]. Balasundaram et al. described a patient that had a spontaneous vaginal delivery when sedated with VV ECMO at 26 weeks and 2 days of gestation with a subsequent satisfactory recovery [17].

The vertical transmission of SARS-CoV-2 has been rarely reported [18,19,20]. Nonetheless, more extensive data are needed to firmly rule out transplacental vertical transmission. A recent study analyzed the placentas of mothers who had suffered severe COVID-19 infection requiring ECMO. The authors found thrombosis in the basal plate’s spiral arteries, intramural endovascular trophoblasts in the third-trimester placentas, subchorionic hematoma, and chronic chorioamnionitis in the fetal membranes [21].

Pregnant women are usually younger and healthier than the general population [22]. This may contribute to the survival of pregnant patients with ECMO. According to the literature, the most common side effects in pregnant patients related to ECMO are bleeding, nosocomial infections, limb ischemia, and venous thromboembolism [4].

The decision of which patients are candidates for an ECMO implant is complex and should be determined by a multidisciplinary team [23]. If the candidate is a pregnant woman with severe COVID-19, it requires an even more thorough decision process. Therefore, ECMO (both VV and AV) should be used with caution in pregnant patients in specialized centers, with multidisciplinary management that includes an obstetric follow-up [11]. In the presented case, the decision that was determined was to place VV ECMO after assessing the state of refractory hypoxemia and the lack of response to the rest of the treatments. Published case reports of pregnant women with severe COVID-19 disease who were supported with VV ECMO describe satisfactory outcomes [4,12,24,25,26].

Peripartum patients needing VV ECMO had a lower mortality rate than those receiving VA ECMO, while those who needed ECMO for respiratory indications had a higher mortality rate than those with cardiac indications [27]. In the presented patient, the double femorojugular cannula technique was used. The blood is drained from the inferior vena cava with a multiperforated cannula inserted through the femoral vein and reinfused already oxygenated in the right atrium with a cannula in the right internal jugular [6]. In our hospital, the double-lumen Avalon is not routinely used because the implant is more complex, the flows are more limited, and there is a higher possibility of cardiac injury. Additionally, the cannula’s nanopositioning must be checked more frequently, preferably with a transesophageal ultrasound. In this case, the patient had COVID-19 and was strictly isolated. This complicated the close monitoring.

In our patient, ECMO began with flows of 4.2 L/min, increasing during the first days to 5.5 L/min to achieve proper oxygenation, considering that the patient was a pregnant woman. Protective pulmonary ventilation was instituted, pending the recovery of lung function.

It should be noted that the implantation of ECMO entails the need for anticoagulation. In the presented patient, two prothrombotic factors were added: pregnancy and COVID-19. Thus, anticoagulation monitoring had to be strict. With the implantation of ECMO, unfractionated heparin was used with TCA controls every two hours at first and, subsequently, every four hours, looking for TCA ranges between 160 and 180 s. In this case, there were no bleeding or thrombotic complications during ECMO use, unlike other studies, in which, unlike what might be expected, the most significant complication was bleeding [5,27,28].

COVID-19 vaccines are safe and effective in the periconceptional period, throughout gestation, and during breastfeeding [29,30,31,32]. The mRNA vaccines do not contain live viruses and do not cause genetic modifications [33]. Additionally, maternal–fetal benefits have been associated with COVID-19 vaccination, such as decreased severe COVID-19 disease, with an 80% lower risk of developing pneumonia and requiring hospital admission, and the placental transmission of protective antibodies to the fetus [29,34]. A recent analysis of six European countries showed that the vast majority of pregnant women admitted to the ICU due to severe COVID-19 during the last half of 2021 was unvaccinated [35]. The presented patient, who required ECMO due to severe COVID-19, was not vaccinated. Additionally, the predominant SARS-CoV-2 variant in Spain at the time of her hospital admission was the pre-alpha variant [36]. This variant was associated with a lower viral load decline rate due to changes in posterior variants’ viral dynamics related to the evolution of SARS-CoV-2 toward a more rapid viral clearance [37]. A recent study stated that when the pre-alpha strain was predominant in Spain, all pregnant patients with SARS-CoV-2 infection in their cohort were unvaccinated, the rate of hospital admission was 6.6%, and progression to severe COVID-19 disease was 2.2% [29]. Several studies revealed that COVID-19 vaccination should be strongly recommended to pregnant women to decrease the risk of severe COVID-19 [29,30,31,38].

## 4. Conclusions

Severe COVID-19 in pregnancy may lead to the requirement for ECMO. This therapy should be administered in specialized hospitals using a multidisciplinary approach. COVID-19 vaccination should be strongly recommended to pregnant women to decrease the risk of developing severe COVID-19.

## Figures and Tables

**Table 1:** Gasometric data during ICU admission.

Days of ICU Admission	Ventilatory Support	FiO_2_	ECMO FiO_2_	PaO_2_/FiO_2_	PCO_2_
1–3	High-flow nasal cannula	80%		139 mmHg	34.7 mmHg
4–7	Invasive mechanical ventilation	100%		72.2 mmHg	45.2 mmHg
8	ECMO (1d)	60%	100%	101 mmHg	42.8 mmHg
9	ECMO (2d)	60%	100%	272 mmHg	44.7 mmHg
15 (tracheostomy)	ECMO (7d)	60%	80%	252 mmHg	33.7 mmHg
17	ECMO (9d)	50%	80%	303 mmHg	33.7 mmHg
21	ECMO WITHDRAWAL (14d)	50%	Without ECMO gas blender	382 mmHg	37 mmHg
23	Invasive mechanical ventilation	50%		232 mmHg	35.8 mmHg
24	High-flow tracheal oxygenation	50%		196 mmHg	37 mmHg
33 (ICU discharge)	Venturi mask	28%		409 mmHg	36.9 mmHg

## Data Availability

Data regarding the case report were obtained from the clinical program of La Fe University and Polytechnic Hospital. More specific data are not publicly available due to privacy, although may be available on request to the corresponding author.
